# Evaluation of Antimicrobial Properties and Potential Applications of *Pseudomonas gessardii* M15 Rhamnolipids towards Multiresistant *Staphylococcus aureus*

**DOI:** 10.3390/pharmaceutics15020700

**Published:** 2023-02-19

**Authors:** Carmine Buonocore, Rosa Giugliano, Gerardo Della Sala, Fortunato Palma Esposito, Pietro Tedesco, Veronica Folliero, Massimiliano Galdiero, Gianluigi Franci, Donatella de Pascale

**Affiliations:** 1 Department of Ecosustainable Marine Biotechnology, Stazione Zoologica Anton Dohrn, Via Ammiraglio Acton, 55, 80133 Naples, Italy; 2 Institute of Biochemistry and Cell Biology, National Research Council, 80131 Naples, Italy; 3 Department of Experimental Medicine, University of Campania “Luigi Vanvitelli”, 80138 Naples, Italy; 4 Department of Medicine, Surgery and Dentistry “Scuola Medica Salernitana”, University of Salerno, 84081 Baronissi, Italy

**Keywords:** rhamnolipids, biosurfactants, MRSA, *S. aureus*, biofilm, SEM, wound dressing

## Abstract

*Staphylococcus aureus* is a Gram-positive opportunistic human pathogen responsible for severe infections and thousands of deaths annually, mostly due to its multidrug-resistant (MDR) variants. The cell membrane has emerged as a promising new therapeutic target, and lipophilic molecules, such as biosurfactants, are currently being utilized. Herein, we evaluated the antimicrobial activity of a rhamnolipids mixture produced by the Antarctic marine bacterium *Pseudomonas gessardii* M15. We demonstrated that our mixture has bactericidal activity in the range of 12.5–50 µg/mL against a panel of clinical MDR isolates of *S. aureus*, and that the mixture eradicated the bacterial population in 30 min at MIC value, and in 5 min after doubling the concentration. We also tested abilities of RLs to interfere with biofilm at different stages and determined that RLs can penetrate biofilm and kill the bacteria at sub-MICs values. The mixture was then used to functionalize a cotton swab to evaluate the prevention of *S. aureus* proliferation. We showed that by using 8 µg of rhamnolipids per swab, the entire bacterial load is eradicated, and just 0.5 µg is sufficient to reduce the growth by 99.99%. Our results strongly indicate the possibility of using this mixture as an additive for wound dressings for chronic wounds.

## 1. Introduction

Multidrug resistance (MDR) in bacteria is a global health concern. Reports estimate 29,000 deaths by MDR bacteria per year in the USA, and 33,000 in Europe [1].

The ESKAPE (*Enterococcus faecium*, *Staphylococcus aureus*, *Klebsiella pneumoniae*, *Acinetobacter baumannii*, *Pseudomonas aeruginosa*, and *Enterobacter* species) pathogens represent a group of MDR bacteria, a leading cause of nosocomial infection for which new effective antibiotics are lacking [2]. Among them, *S. aureus* plays a relevant role as it is responsible for numerous diseases, such as skin infections, endocarditis, pneumonia, and septicemia [3]. *S. aureus* is an opportunistic Gram-positive human pathogen carried by 20–40% of the human population [4,5]. Most of the reported problems are caused by methicillin-resistant *S. aureus* (MRSA), characterized by resistance to beta-lactam antibiotics, leading to infections that are difficult to treat and associated with increased mortality [6]. Due to its pathogenic potential, many other *S. aureus* strains of clinical relevance, such as macrolide-resistant *S. aureus* and vancomycin-resistant *S. aureus* (VRSA), are also associated with increased mortality rates [7]. Moreover, *S. aureus*, particularly MRSA, is the leading agent in wound infections, also playing a pivotal role in the chronicization of infected wounds [8,9]. Indeed, *S. aureus* can act as a pioneer, producing several virulence factors that facilitate cell adhesion, such as fibrins that form a scaffold for the production of bacterial biofilm [10]. Since classical antimicrobial approaches are not successful in eradicating infections where bacteria are quiescent, such as in biofilm, it is hard to eradicate chronic wounds [11].

In this scenario, innovative approaches could represent an alternative, such as additives or adjuvants for wound dressing. Moreover, as the probability of encountering *S. aureus* resistant to most (or all) of the conventional antibiotics is increasing, there is also an urgent need for new antimicrobial targets [5,12]. Cell membranes could be a suitable target as they are essential in all metabolic statuses. Regrettably, membrane-active drugs are generally lipophilic and commonly associated with toxicities [5]. However, some membrane-active antibiotics, such as the lipopeptides daptomycin, telavancin, oritavancin, and dalbavancin reached the market, indicating that selectivity for bacterial membranes is achievable [11].

**Figure 1:**
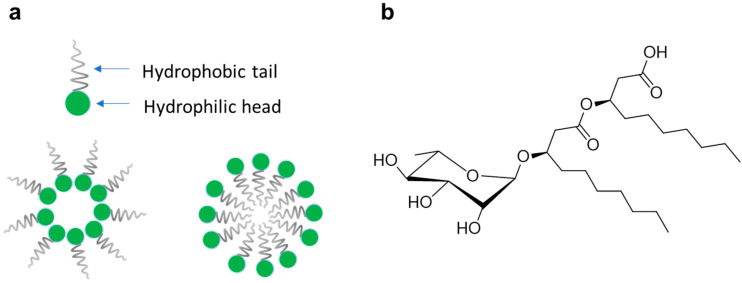
Biosurfactant overview (**a**); and mono-RL general structure (**b**).

Some of the molecules mentioned above are biosurfactants. They are natural products with an amphiphilic nature, able to lower the surface tension between two phases (Figure 1a) [13]. One of the most well-known classes of biosurfactants is represented by rhamnolipids (RLs), a family of glycolipids produced by many Gram-negative bacteria, consisting of one (mono-RL) or two (di-RL) rhamnose units linked through glycosidic linkage to one or two β-hydroxy fatty chains (Figure 1b) [14]. The fatty acid chains could be saturated or unsaturated, with a number of carbons ranging from 8 to 24, although fatty acids with C_8_-C_14_ are the most common [14,15]. RLs possess many bioactivities, such as anticancer, antifungal, antibacterial, antioxidant, antibiofilm, and antiviral activities [16,17,18,19,20]. Moreover, they are currently used as additives in commercial formulations of cosmeceutical products (i.e., Evonik and TeeGene) [21,22].

More than 60 RLs have been reported to date. They are mainly produced by *Pseudomonas aeruginosa*, while other producing genera include *Nocardiopsis*, *Acinetobacter*, *Enterobacter*, and *Burkholderia* [23].

In previous work, we characterized a mixture of RLs produced by the Antarctic marine bacterium *Pseudomonas gessardii* M15, highlighting its antimicrobial activity against a panel of Gram-positive bacteria [24]. In follow-up work, we proved the ability of this mixture (M15RL) to inactivate viruses from the *Herpesviridae* and *Coronaviridae* families. In particular, we demonstrated that M15RL can inactivate enveloped viruses on treated surfaces [25].

In this study, we expanded the panel of tested *S. aureus* strains, achieving promising antibacterial and antibiofilm activities of M15RL against reference and drug-resistant strains, including MRSA. Consequently, we investigated the possible application of M15RL as an additive for wound dressing. For this purpose, we explored the ability of M15 to functionalize textiles, preparing cotton swabs coated with M15RL as a proof of concept. The results showed M15RL had high effectiveness in functionalizing textiles and thus its potential utilization for the treatment of chronic wounds. Finally, to demonstrate its possible industrial processing, we assessed the stability of the mixture in terms of antimicrobial activity at high temperatures and in a wide pH range.

## 2. Materials and Methods

### 2.1. Rhamnolipids Mixtures

The investigated rhamnolipids mixture was obtained from *P. gessardii* M15 (M15RL), as previously described [24]. Briefly, *P. gessardii* M15 was cultivated in TYP (16 g/L bacto-tryptone, 16 g/L yeast extract, 10 g/L NaCl) at 20 °C for 5 days. After that period of incubation, the supernatant was collected by centrifugation, filtered with a 0.22 µm filter, and extracted twice with ethyl acetate 1:1 *v*/*v*. The extract was further processed by solid-phase fractionation (SPE) through a CHROMABOND C18 reversed-phase SPE column (MACHEREY-NAGEL GmbH & Co. KG, Duren, Germany). Briefly, the crude extract was resuspended in methanol (MeOH) and loaded on the pre-activated SPE column, which was successively washed with two column volumes of H_2_O. Then, three different fractions were obtained by flushing the SPE column with two column volumes of 65% MeOH/H_2_O (*v*/*v*), 80% MeOH/H_2_O (*v*/*v*), and MeOH, respectively. The fraction eluted with 80% MeOH/H_2_O (*v*/*v*)contained a mixture of mono-rhamnolipids and was named M15RL. This mixture was utilized in all the tests presented in this work [24,25].

Two commercial mixtures of RLs, produced by *P. aeruginosa*, were purchased from Sigma-Aldrich (Sigma-Aldrich, Darmstadt, Germany). Both mono- and di-rhamnolipids are present in these mixtures, but one is mono-rhamnolipid dominant (MDRL), while the other is di-rhamnolipid dominant (DDRL).

### 2.2. Liquid Chromatography-High Resolution Tandem Mass Spectrometry Analysis of M15RL and Commercial RLs Mixtures

The RL mixtures were dissolved in MeOH at a concentration of 1 mg/mL for Liquid Chromatography—High-Resolution Tandem Mass Spectrometry analyses (LC-HRMS/MS), as previously reported [26]. Chemical profiling was performed using a Thermo LTQ Orbitrap XL high-resolution ESI mass spectrometer equipped with a Thermo U3000 HPLC system and a 5-μm Kinetex C18 column (50 × 2.10 mm) [27]. The column was eluted at room temperature at 200 μL/min with H_2_O (supplemented with 0.1% HCOOH) and CH_3_CN, using gradient elution. The gradient program was set as follows: 25% CH_3_CN for 3 min, 25−80% CH_3_CN for 60 min, and 95% CH_3_CN for 7 min. Mass spectra were recorded in the positive ion detection mode (mass accuracy ≤ 3 ppm). MS parameters were set as follows: spray voltage of 4.8 kV, capillary temperature of 285 °C, sheath gas rate of 32 units N_2_ (ca. 150 mL/min), auxiliary gas rate of 15 units N_2_ (ca. 50 mL/min). MS/MS data were collected in the data-dependent acquisition mode to fragment the five most intense ions of a full-scan mass spectrum. The *m/z* range for the data-dependent acquisition was set between 150 and 2000 Dalton. HRMS/MS scans were obtained with CID fragmentation, setting an isolation width of 2.0, normalized collision energy of 35, activation Q of 0.250, and an activation time of 30 ms. The fragment ion generated from in-source fragmentation of the detected rhamnolipids, after the loss of a rhamnose or di-rhamnose unit, was selected for relative quantification of each congener. Areas of the in-source fragments were calculated using the Thermo Xcalibur software (v. 2.2, Thermo Fisher Scientific, Waltham, MA, USA). The relative abundance of each peak was calculated as the percentage of the single rhamnolipid fragment peak divided by the sum of all the rhamnolipid fragment peak areas.

### 2.3. Antibacterial Assay

The antimicrobial activities were assessed on a panel of both reference and clinically isolated *Staphylococcus aureus* strains, as follows: *S. aureus* ATCC^®^ 6538™, *S. aureus* ATCC^®^ 6538p™, methicillin-sensitive *S. aureus* (MSSA), methicillin-resistant *S. aureus* (MRSA), β-lactamase-producing *S. aureus* (β-LPSA), macrolide-lincosamide-streptogramin B-resistant *S. aureus* (MLSB), quinolone-resistant *S. aureus* (QRSA), vancomycin-resistant *S. aureus* (VRSA) [28]. First, each strain was plated on Luria-Bertani (Lennox) (CondaLab, Madrid, Spain) agar (LBA) plates and incubated overnight at 37 °C. Then, 2–3 fresh colonies were transferred to 3 mL of Muller Hinton (MH) broth (HiMedia, Einhausen, Germany) and incubated in agitation overnight at 37 °C. The day after, the bacterial suspensions were transferred to fresh MH medium and further incubated until they reached the turbidity of 0.5 McFarland. Finally, serial dilutions of the inoculum were performed to obtain a final bacterial concentration of about 5 × 10^5^ CFU/mL. Stock solutions of the samples were prepared by dissolving M15RL, MDRL, and DDRL in dimethylsulfoxide (DMSO) at 10 mg/mL. The tests were conducted with the broth microdilution method to evaluate the antimicrobial potential of these mixtures. Briefly, 4 µL of each sample was dispensed into 200 µL of MH medium in a microtiter plate and twofold serially diluted. Then 100 µL of bacterial suspension was inoculated into the broth (~5 × 10^4^ CFU/well) and incubated statically for 18h at 37 °C. Vancomycin and DMSO (2% *v*/*v*) represented the positive and negative controls, respectively.

### 2.4. MIC and MBC

The minimal inhibitory concentration (MIC) was assessed by comparing the optical density (OD) of the treated strain with the control, untreated, recorded with a Tecan plate reader (Tecan, Männedorf, Switzerland) at 600 nm. After the determination of MIC, the minimal bactericidal concentration (MBC) was calculated. Briefly, 100 µL from ½ × MIC, 1 × MIC, and 2 × MIC dilutions were subcultured onto fresh LBA plates for 24 h at 37 °C. After the incubation, the MBC was recorded as the lowest concentration that prevented the growth of colonies.

### 2.5. Time-Kill

After determining MBC, a time-kill assay of M15RL was performed against *S. aureus* 6538. The experiment was conducted in the same condition as the microdilution assay, recording the colonies forming unity (CFU) at defined intervals (0.5, 1, 2, 3, 6, and 24 h). Briefly, for each time point, 10 µL of bacterial suspension was taken from the ½ × MBC, 1 × MBC, and 2 × MBC wells, serially 10-fold diluted in Dulbecco’s phosphate buffered saline (PBS), and then each dilution was plated onto fresh LBA plates [29]. After 24 h of incubation at 37 °C, emergent colonies were counted, and the CFU/mL were compared with the ones of the bacterial control (untreated).

### 2.6. Scanning Electron Microscopy

The action of M15RL on the *S. aureus* 6538 morphology was investigated by scanning electron microscopy (SEM). Before preparation for microscopic observation, *S. aureus* 6538 was processed as described in the antimicrobial assay. After 18 h at 37 °C, 200 μL of the bacterial suspension treated with M15RL at ½ × MIC, 1 × MIC, 2 × MIC, and 4 × MIC was taken and centrifugated at 2500× *g* for 15 min. For the observation, the cells were treated with little modifications, as described before by Sotirova [30]. Briefly, the cells were washed in 0.1 M Na cacodylate buffer (CB), fixed for 4 h at 4 °C in 4% glutaraldehyde in CB, resuspended in CB and poured on a 0.4 µm filter, washed in CB, and post-fixed in the dark for 1 h at 4 °C in 1% OsO_4_ in CB. The filters were washed in CB, successively in MilliQ water, and then dehydrated in graded ethanol series. Finally, the samples were vacuum coated with gold. Observations were made on JEOL 6700 FE SEM (JEOL, Tokyo, Japan).

### 2.7. Antibiofilm Activity

The antibiofilm activity against *S. aureus* 6538 and MRSA was investigated in the different steps of biofilm formation and maturation by four assays, as follows:

#### 2.7.1. Initial Cell Attachment Assay

The initial bacterial attachment to the substrate was investigated with some modifications from [31]. *S. aureus* strain 6538 and MRSA were grown overnight in MH. Absorbance at OD_600_ was measured and adjusted to 0.1 OD (~8 × 10^7^ CFU/mL) in MH supplemented with 1% (*v*/*v*) glucose. The compounds and bacteria were added in each well of the 96-well microplate at the final volume of 200 µL. Untreated bacteria were used as the positive control. Then, the plates were incubated at 37 °C for 1 h. Media were discarded, and the wells were washed two times with PBS to remove any non-adherent planktonic cells. Additionally, 180 µL of fresh MH and 20 µL of MTT 3-(4,5-dimethylthiazol-2-yl)-2,5-diphenyl tetrazolium bromide solution were added to each well, and the plates were incubated for 2 h at 37 °C. Then, the cellular adhesion was measured through a colorimetric assay. Absorbance at 570 nm was measured, and each experimental well was normalized to the control (untreated).

#### 2.7.2. Biofilm Inhibition Assay

The inhibitory effect of biofilm formation was determined by crystal violet (CV) dye. First, 200 µL of an overnight *S. aureus* 6538 and MRSA culture was adjusted to 0.1 OD_600_ and dispended in a 96-well microtiter plate. The M15RL (at different concentrations) was added to the wells, and the plates were incubated overnight at 37 °C. After, the medium was discarded, and the plates were washed with PBS twice and dried. The plates were stained with aqueous crystal violet (0.05%), followed by incubation for 30 min, at room temperature (RT). Then, the CV stain was removed, and the plates were washed with water. The biofilm-adhered stain was resolubilized in ethanol, and the plates were incubated at RT for 30 min. Biofilm inhibition was quantified by measuring OD_570_ [32]. The inhibition was calculated by comparing absorbance at 570 nm of the biofilm in the control with the absorbance of the treated condition.

#### 2.7.3. Biofilm Degradation

The degradation activity of the biofilm was evaluated with some modification from [33]. Briefly, *S. aureus* 6538 and MRSA were inoculated into TSB for 24 h at 37 °C. After the incubation, the inoculum was adjusted to 0.1 OD_600_. Then, 100 µL of the bacterial suspension was transferred to a 96-well plate and incubated at 37 °C for 72 h, refreshing the plate with new media every 24 h. The negative control was represented by wells filled with 100 μL of the medium. After the incubation, the plate was washed three times with distilled water to remove the floating, non-adhesive bacteria. Then, 100 µL of fresh TSB containing M15RL at different concentrations was added to each well, and the plate was incubated at 37 °C O/N. The plate was washed three times with distilled water, and wells were rinsed with 110 µL of 0.05% (*v*/*v*) CV and incubated in orbital agitation for 40 min at RT. Then, the plate was washed with distilled water to remove the excess dye. Finally, the CV was solubilized by incubation with 110 µL of ethanol in orbital agitation for 40 min, at RT. Biofilm biomass was measured by recording the absorbance at 570 nm with the microplate reader TECAN SUNRISE (Tecan Group Ltd., Männedorf, Switzerland).

#### 2.7.4. Biofilm Penetration

To evaluate the metabolic activity of *S. aureus* cells in the biofilm, 100 µL of overnight *S. aureus* 6538 and MRSA suspensions at 0.1 OD was dispensed into a 96-microtiter plate. The plate was incubated for 48 h at 37 °C to allow the formation of a mature and robust biofilm, as described above. Subsequently, the medium was gently removed, and the biofilm was gently washed twice with PBS. Then, the biofilm was treated for 4 h and 24 h with different concentrations of M15RL from 100 to 1.56 µg/mL dissolved in fresh MH medium. After the incubation, the supernatant was removed, and the biofilm was washed again with PBS. Finally, 100 µL of MTT (5 mg/mL) was added into each well, and the plate was incubated for 2 h at 37 °C. The percentage of metabolically active cells within the biofilm was calculated by recording the absorbance at 570 nm with the microplate reader TECAN SUNRISE (Tecan Group Ltd., Männedorf, Switzerland).

### 2.8. Cotton Swabs

The antibacterial activity of textiles functionalized with M15RL was investigated by using a cotton swab as a model. First, the volume of liquid taken from each swab was calculated (ca. 80 µL) out of 10 replicates. Then, we calculated the total quantity of RLs present on each swab, multiplying the stock solution concentration by the obtained volume. Stock solutions of the mixture were made by dissolving M15RL at 50, 25, 12.5, and 6.25 μg/mL in water. The bacterial suspension of *S. aureus* 6538 was prepared as follows: 2–3 colonies from a fresh plate were dispensed in 3 mL of MH and incubated in agitation overnight at 37 °C. Then, the inoculum was refreshed with new MH and incubated at 37 °C until it reached 0.5 McFarland. Finally, the bacterial suspension was adjusted to 10^5^ CFU/mL. The swabs were sterilized by autoclaving them for 40 min. Functionalized cotton swabs were prepared by soaking them in the stock solutions and letting them dry sterilely in the laminar airflow cabinet. Swabs soaked in sterile water were utilized as negative controls. Once dry, cotton swabs were soaked in the *S. aureus* 6538 suspension, transferred to 15 mL sterile conical tubes, and incubated statically for 20 h at 37 °C. After the incubation, the swabs were transferred to new sterile conical tubes, rinsed with 1 mL of PBS, and vortexed for 30 s, twice. Then, 100 μL from each condition was serial diluted, plated on agar plates, and statically incubated at 37 °C. After 18 h of incubation, the CFUs of the treated conditions were counted and compared to the control (untreated).

### 2.9. Hemolytic Assay

The hemolytic activity of M15RL was determined using fresh human erythrocytes of blood group 0 from healthy volunteers. Briefly, 10 mL of blood suspension was centrifuged at 500× *g* for 5 min, and the plasma level was marked, then gently aspirated and discarded. The hematocrit tube was filled with a solution of 150 mM NaCl to the original plasma level and washed three times. Then, erythrocytes were diluted in PBS to 1:50, and 180 µL was added into the wells of a U-shaped bottom 96-well plate. Subsequently, 20 µL of M15RL was added at different concentrations (6 to 100 µg/mL) and incubated for 1 h at 37 °C. PBS and 0.1% (*v*/*v*) Triton X-100 represented negative and positive controls, respectively. Finally, the plate was centrifuged for 5 min at 500 g, then the supernatant was transferred into a new plate, and the absorbance of the released hemoglobin was recorded at 540 nm using the microplate reader TECAN SUNRISE (Tecan Group Ltd., Männedorf, Switzerland) [34].

The percentage of hemolysis was calculated using the equation:

% of hemolysis = [(Abs sample)/(Abs Triton)] × 100

### 2.10. Chemical and Physical Stress

The resistance of M15RL to high temperatures was evaluated at two different temperatures: 80 °C and 121 °C. Three 1 mL stocks of the mixture at 10 mg/mL in DMSO were prepared. One was incubated in a thermostatic water bath at 80 °C, and aliquots of 8 μL were taken at scheduled times (0, 1, 2, 4, 6, 8, and 24 h), while the other two were autoclaved at 121 °C for 20 and 40 min, respectively. To assess the stability, all the obtained aliquots were tested for antimicrobial activity against *S. aureus* 6538, as described above. MH at different pH values (4, 5, 6, 7, 8, 9, and 9.8) was prepared to evaluate the effects of chemical stress. The pH dependence of M15RL antimicrobial activity was investigated using MH at values ranging from 5 to 9 as growing media to determine the MICs against *S. aureus* 6538. Cultures at the same pH without M15RL were utilized as controls. To test the mixture’s stability to different pH levels, stock solutions of M15RL at 10 mg/mL were prepared in MH at a pH from 4 to 9.8 and incubated at 4 °C for up to 2 months. Then, an antimicrobial assay was performed against *S. aureus* 6538 with little modification. Briefly, at the selected time, 1 μL from each condition was dispensed into 200 µL of fresh MH in a microtiter plate and twofold serially diluted. After that, the assay was performed as described above. Additionally, 1 μL of MH at the appropriate pH without M15RL was used as the control.

### 2.11. Statistical Analysis

Graphs and one-way ANOVA followed by the Dunnett’s multiple comparisons tests were generated with GraphPad Prism 9 for Windows ver. 9.0.0 (GraphPad Software, San Diego, CA, USA, www.graphpad.com).

## 3. Results

### 3.1. Chemical Characterization of M15RL and Commercial RLs Mixtures

The M15RL and the commercial mono-rhamnolipid dominant and di-rhamnolipid dominant mixtures (MDRL and DDRL, respectively) were analyzed individually by LC-HRMS/MS. The analysis of mass tandem spectra allowed for the identification of rhamnolipid congeners present in the three different mixtures, as reported in Table 1.

The product ion spectra of the [M+H]^+^ and/or [M+Na]^+^ ions of rhamnolipids were dominated by fragments generated by neutral losses of (a) the rhamnose residue (i.e., C_6_H_10_O_4_, 146.0579 amu) and (b) the terminal β-hydroxy fatty acyl group as an α-β unsaturated fatty acid (Figure S4). Sequential losses of two rhamnose residues were observed for di-rhamnolipids. Basically, the diagnostic fragment ion arising from the cleavage of the ester linkage between the two β-hydroxy fatty acids was used for the structural prediction of the different congeners in each mixture [24].

As compared to MDRL and DDRL mixtures from *P. aeruginosa,* the M15RL mixture from *P. gessardii* M15 was composed exclusively of mono-rhamnolipids. In addition, M15RL showed the higher relative abundance of mono-rhamnolipids bearing β-hydroxy fatty acids with chain lengths greater than C10.

### 3.2. Antimicrobial Activity of M15RL and Commercial RLs Mixtures

To compare the antimicrobial activities of M15RL with the commercially available RLs mixtures MDRL and DDRL, a broth microdilution assay was performed against a panel of reference and clinical strains of *S. aureus* (Table 2). M15RL showed the best results, with a minimal inhibitory concentration (MIC) of 50 µg/mL against VRSA and values ranging from 12.5 to 25 µg/mL against the other strains. Among the commercial RLs, the MDRL mixture showed higher antimicrobial activity than DDRL. In fact, DDRL showed no activity towards MRSA, β-LPSA, QRSA, and VRSA at the investigated concentrations, while MDRL inhibited the growth of all the strains except for MRSA and VRSA.

Subsequently, we also assessed the MBC of the mixtures against all the *S. aureus* strains. Results showed that, except for MSSA and QRSA, for which MBC is equal to 2 × MIC, M15RL is bactericidal at the MIC concentration. On the other side, MDRL is bactericidal at 2 × MIC against SA6538, β-LPSA, and MLSB, being bacteriostatic on the other strains. DDRL showed bactericidal activity only against SA6538 at the MIC value. All these results are summarized in the table below.

**Figure 2:**
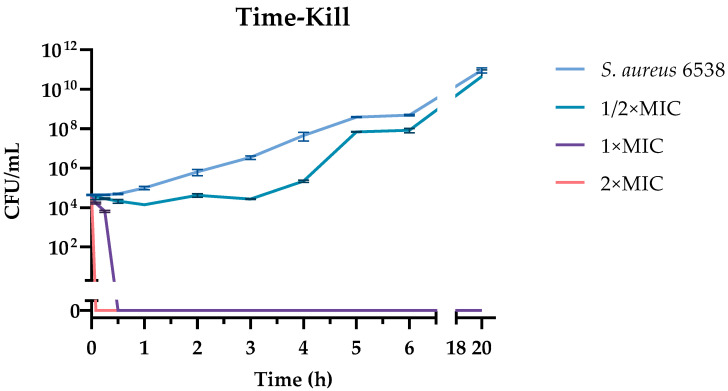
Time-kill assay of M15RL against 6538. At the MIC concentration, M15RL was able to completely eradicate the bacterial load in 30 min. It requires just 5 min at 2 × MIC.

Once we established the bactericidal effect, we monitored the time required for M15RL to kill *S. aureus* 6538 at the MIC in a time-course experiment. The time-kill assay revealed that M15RL eradicated the entire bacterial population after 5 min at 2 × MIC, and after 30 min at MIC (Figure 2). On the other side, the mixture at ½ × MIC is mainly bacteriostatic in the first 4 h, and the population of *S. aureus* quickly reaches the control (untreated) after 6 h.

### 3.3. Visualization of M15RL Activity on S. aureus

**Figure 3:**
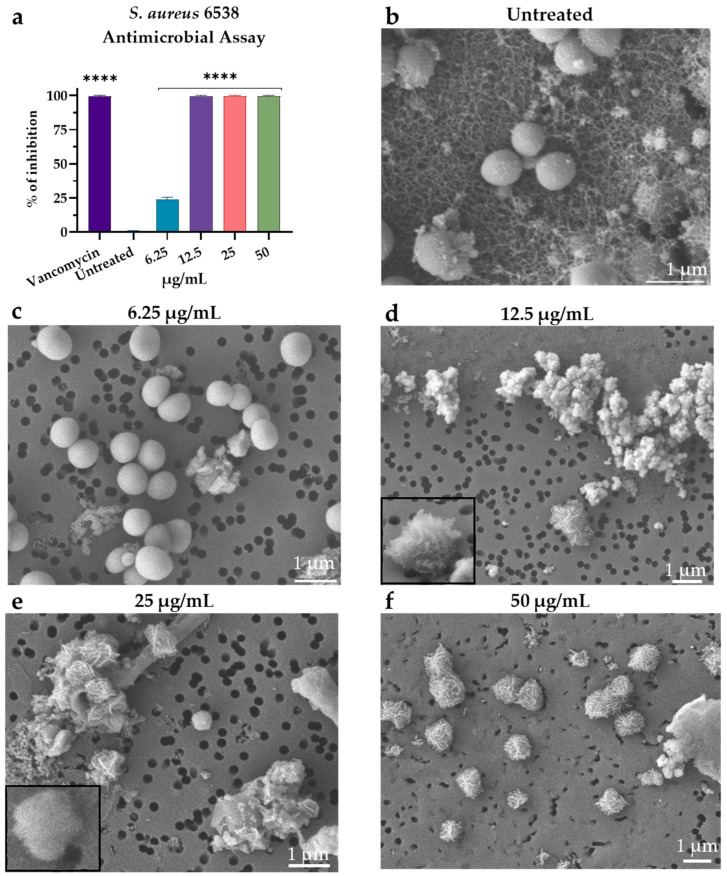
SEM visualization of *S. aureus* cells treated with M15RL in the microtiter assay conditions (**a**). In control (**b**) and in the sample treated with M15RL at concentration lower than MIC (**c**), *S. aureus* cells present a healthy morphology. Differently, when treated with MIC concentration (**d**) and over (**e**,**f**), cells lose their integrity, and cytoplasmatic material is reversed outside the cells. Statistical analyses were determined by ANOVA with Dunnett’s test for multiple comparisons. Significances are referred to the untreated bacterium. **** *p* < 0.0001.

The activity of M15RL against *S. aureus* 6538 was further investigated by SEM microscopy. *S. aureus* cells treated with M15RL below the MIC concentration (Figure 3c) are similar to the control (untreated), showing a round shape and normal morphology. Increasing the concentration of M15RL to the MIC and above, the accumulation of intracellular material due to cellular damage can be observed outside the cells (Figure 3d–f). Moreover, cells lose their structural integrity (Figure 3e).

### 3.4. Effect of M15RL on S. aureus Biofilm

**Figure 4:**
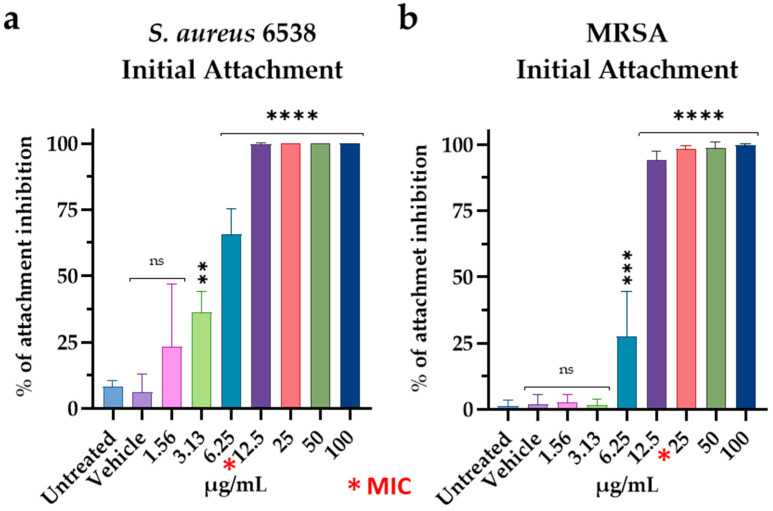
M15RL interference with the attachment of (**a**) *S. aureus* 6538 and (**b**) MRSA to the substrate. Statistical analyses were determined by ANOVA with Dunnett’s test for multiple comparisons. Significances are referred to the untreated bacteria. **** *p* < 0.0001, *** *p* < 0.0002, ** *p* < 0.0021, ns (not statistically significant).

The stages of biofilm formation have been deeply studied and can be divided into three major events: initial attachment, biofilm maturation, and dispersal [35]. During the initial attachment, the microbial cells irreversibly bind to the surface. Following the attachment, bacteria start the production of an extracellular matrix and the biomass accumulates with the formation of a mature biofilm [35]. Finally, the cells within the biofilm can detach and return to a planktonic state through dispersal [36]. Here, we investigate the ability of M15RL to interfere in the first two stages of *S. aureus* 6538 and MRSA biofilm formation and its ability to penetrate or destroy it once formed. In the attachment assay, against *S. aureus* 6538 M15RL exhibited a complete reduction of the attachment at 12.5 µg/mL (MIC) and above, achieving 70% at 6.25 µg/mL (½ MIC) (Figure 4a). Interesting results were also obtained against the MRSA, with 90% of attachment reduction at 12.5 µg/mL (½ MIC), and complete reduction above (Figure 4b).

**Figure 5:**
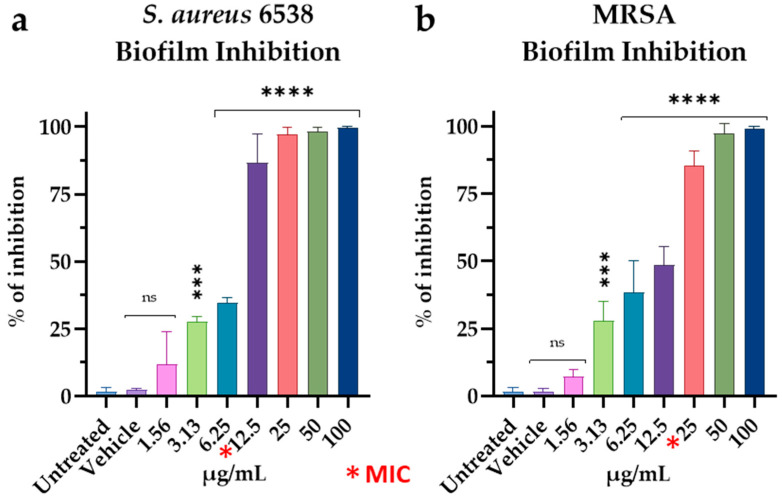
Inhibition of mature biofilm formation against (**a**) *S. aureus* 6538 and (**b**) MRSA. Statistical analyses were determined by ANOVA with Dunnett’s test for multiple comparisons. Significances are referred to the untreated bacteria. **** *p* < 0.0001, *** *p* < 0.0002, ns (not statistically significant).

Matrix accumulation is the second step of biofilm formation. We investigated the ability of M15RL to inhibit the formation of a mature biofilm. M15RL showed 85% reduction at 12.5 µg/mL (MIC) and complete biofilm inhibition at higher concentrations against *S. aureus* 6538 (Figure 5a). Against MRSA, M15RL showed similar activity in terms of MIC concentrations. In fact, the activity accounted to 85% at 25 µg/mL (MIC) (Figure 5b).

**Figure 6:**
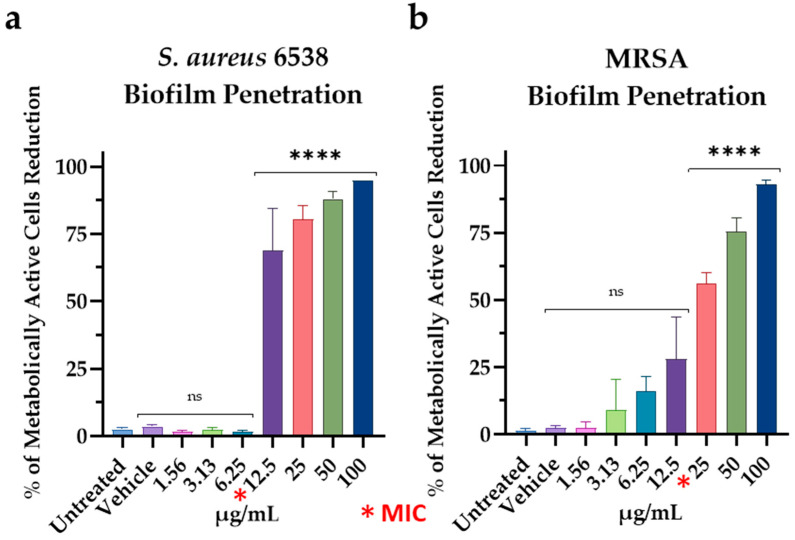
The ability to kill *S. aureus* 6538 (**a**) and MRSA (**b**) bacteria inside the biofilm was evaluated by the biofilm penetration assay. Results are presented as the percentage reduction of metabolically active cells. Statistical analyses were determined by ANOVA with Dunnett’s test for multiple comparisons. Significances are referred to the untreated biofilms. **** *p* < 0.0001, ns (not statistically significant).

The ability of M15RL to penetrate the biofilm and kill cells inside was evaluated by the MTT method (Figure 6a,b). The results significantly showed a reduction of *S. aureus* 6538 and MRSA metabolically active cells when treated with M15RL at 12.5 µg/mL (MIC) and 50 µg/mL (2 × MIC), respectively.

**Figure 7:**
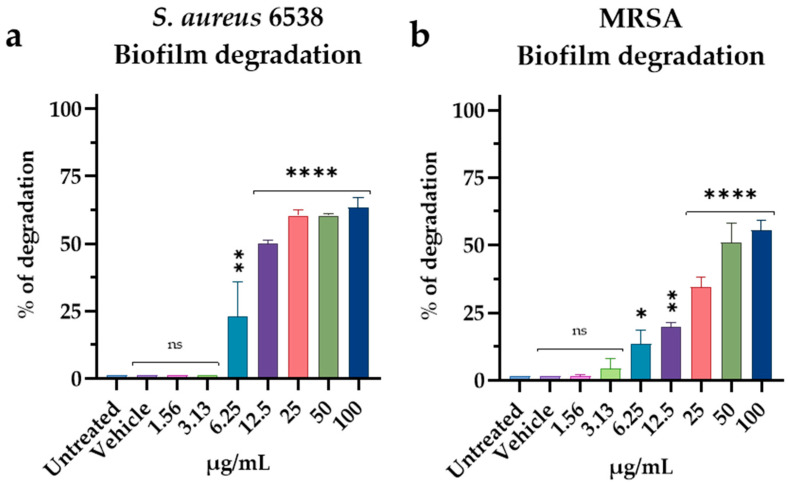
Degradation of mature biofilms produced by (**a**) *S. aureus* 6538 and (**b**) MRSA. Statistical analyses were determined by ANOVA with Dunnett’s test for multiple comparisons. Significances are referred to the untreated bacteria. **** *p* < 0.0001, ** *p* < 0.0021, * *p* < 0.0332, ns (not statistically significant).

The ability to disrupt biofilm was evaluated on a 72 h mature biofilm by CV. Results revealed strong activity against *S. aureus* 6538, with 60% of degradation from 25 to 100 µg/mL and 50% at 12.5 µg/mL (MIC) (Figure 7a). Lower activity was recorded against the MRSA strain, with 50% of degradation at 50 and 100 µg/mL, and 35% of activity at 25 µg/mL (MIC) (Figure 7b).

### 3.5. Antimicrobial Activity of Functionalized Textiles

**Figure 8:**
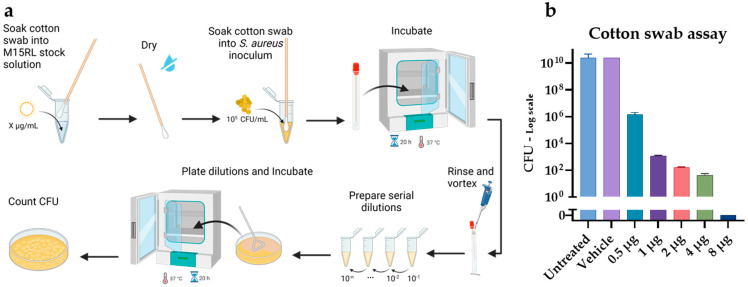
Experimental design of the cotton swab assay (**a**), created with BioRender.com. M15RL-soaked swabs exhibited high antimicrobial activity (**b**).

To assess the ability of M15RL-coated textiles to inhibit *S. aureus* growth, cotton swabs imbibed in decreasing concentrations of the mixture were prepared. The swabs were inoculated with *S. aureus* 6538, and CFU counts were performed. Results showed that the growth was reduced by 99.99% (4-log reduction) in the presence of 0.5 µg of M15RL (Figure 8b). Furthermore, the bacterial load is entirely eradicated when swabs are soaked in a solution of 100 µg/mL (8 µg per swab).

### 3.6. Hemolytic Activity

**Figure 9:**
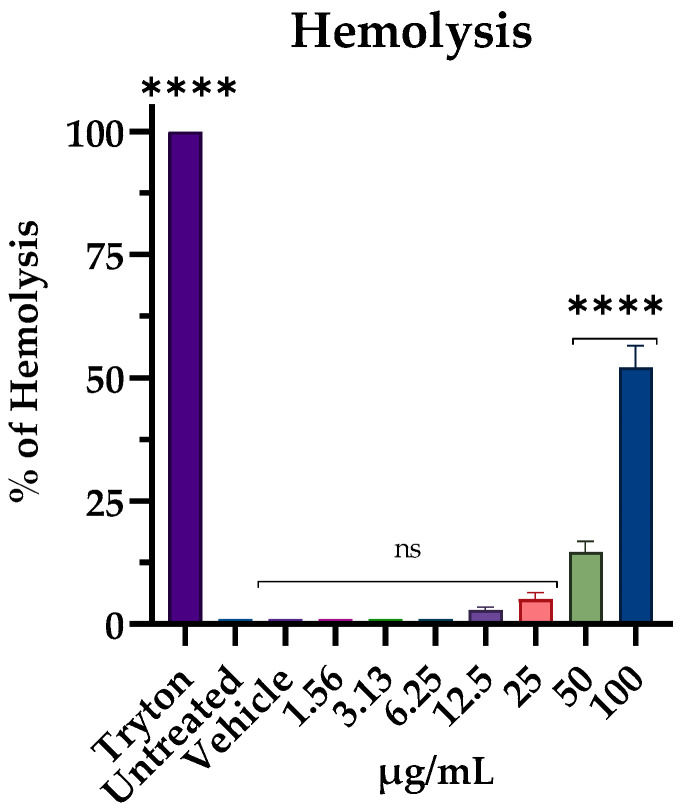
The ability of M15RL to interfere with mammalian cells. Thus, the cytotoxicity was assessed by hemolysis assay. Statistical analyses were determined by ANOVA with Dunnett’s test for multiple comparisons. Significances are referred to the untreated red cells. **** *p* < 0.0001, ns (not statistically significant).

The hemolysis assay evaluated the cytotoxic effect on eukaryotic cells to assess that M15RL was selective towards bacterial membranes. The mixture recorded 50 and 20% of erythrocyte lysis at 100 and 50 ug/mL, respectively. No hemolytic effect was detected at lower concentrations (Figure 9). Triton-X at 0.1% used as positive control showed 100% lysis.

### 3.7. M15RL Stability to Harsh Conditions

**Figure 10:**
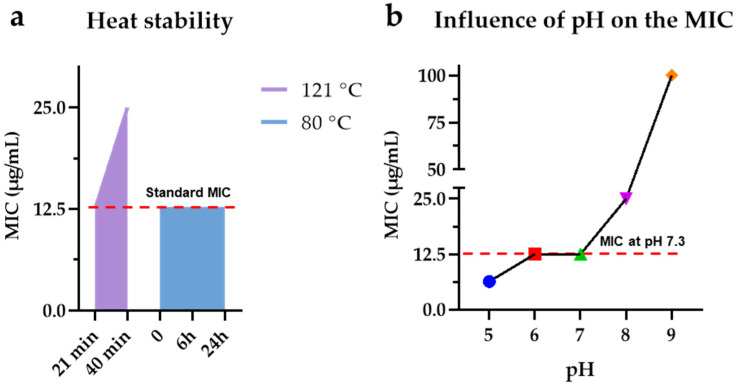
Stability of M15RL to high temperatures and influence of pH on the M15RL MIC. The mixture showed high resistance to high temperatures, being affected only after 40 min at 121 °C (**a**). On the other side, the antimicrobial activity of M15RL is highly affected by the pH of the environment, being less active at pH levels higher than 7 (**b**).

Thermal stability was evaluated by antimicrobial assay against *S. aureus* 6538 after heating M15RL in a water bath (80 °C) and an autoclave (121 °C) at different times before measuring the MIC. Results showed that heating M15RL at 80 °C for up to 24 h, or at 121 °C for 21 min, does not affect its antimicrobial activity. On the contrary, heating the mixture for 40 min at 121 °C lowered its antimicrobial power, increasing the MIC to 25 µg/mL (Figure 10a). The pH stability was investigated by incubating M15RL at different pH values over a long period and assessing the MIC values at physiological/neutral pH (Figure S11). Results showed that incubating M15RL for up to 2 months at pH values ranging from 5.0 to 10.0 does not affect the MIC. Differently, after two months of incubation at pH 4.0, the mixture loses activity, increasing its MIC to 25 µg/mL, probably due to slow partial hydrolysis of the O-glycosidic and/or ester linkages in the acidic environment. In addition, we investigated the antimicrobial activity of M15RL against *S. aureus* 6538, assessing the MIC values at pH values ranging from 5.0 to 9.0. Results revealed that the MIC is highly affected by the pH (Figure 10b). At pH 5.0, the antimicrobial activity is higher than in the standard conditions (pH 7.2): MIC at 6.25 and 12.5 µg/mL, respectively. On the contrary, by increasing the pH, the antimicrobial activity is affected negatively, with MIC increased by two and four times at pH 8.0 and 9.0, i.e., 25 and 100 µg/mL, respectively. The pH values of 6.0 and 7.0 do not affect the activity.

## 4. Discussion

In previous work, we characterized the mixture of RLs produced by *P. gessardii*, M15RL. Furthermore, we demonstrated that M15RL inactivates enveloped viruses belonging to the *Herpesviridae* and *Coronaviridae* families [25]. In this work, we investigated the antibacterial activity of M15RL towards a panel of clinical MDR isolates of *S. aureus*.

To compare the RLs mixture of M15 with others already present on the market, M15RL and the two commercial mixtures (MDRL and DDRL) were tested against *S. aureus* reference (n = 2) and clinical (n = 6) strains. Results revealed that RLs produced by *P. gessardii* have high antimicrobial potential against *S. aureus,* inhibiting all the tested strains. On the contrary, the antimicrobial activity of the tested commercial RLs was significantly lower. The antimicrobial activity differences can likely be attributed to the different RL compositions. In particular, where the mixture mainly comprises di-RLs (DDRL), it shows activity only on the *S. aureus* 6538 strain. On the other hand, the best results were achieved by the mixture composed only of mono-RLs, M15RL. This is in accordance with the literature, suggesting that mono-RLs are more efficient than di-RLs in killing *S. aureus* [37,38]. In addition, M15RL contained a higher relative abundance of mono-RLs bearing β-hydroxy fatty acids with chain lengths greater than C10, as compared to the MDRL mixture. These findings could suggest that the antibacterial activity of mono-RLs increases with the fatty acyl chain length. Indeed, mono-RLs with more than 20 carbons combining both fatty acid chains represent 75% of the total amount of M15RL, while this percentage is just 21% in MDRL. Furthermore, results showed that, except for QRSA and MSSA, the MIC of M15RL corresponds to the MBC, while for MDRL the MBC is at least two times higher. Time-kill results showed how M15RL promptly acts against *S. aureus* 6538. In fact, the mixture eradicated the bacterial population in 5 min at 2 × MIC. These data are even more relevant when compared to the literature, in which a commercial mixture showed no bactericidal activity against *S. aureus* at pH 7 and required 24 h to kill at 4 × MIC with the pH adjusted to 5 [39].

The SEM images allowed us to visualize the effect of M15RL on *S. aureus*. No significant effects were present at concentrations below the MIC. However, starting from the MIC and increasing the concentrations, the cells appear deformed, showing roughness caused by the leakage of intracellular material. The leakage of cytoplasmic materials implies cell lysis or increased cell permeability, which is in agreement with previous reports. In fact, previous works suggested that RLs interact with cell membranes, leading to the release of cytoplasm by pore formation and cell death [39,40,41]. Based on these results, differences in the membrane compositions of the tested *S. aureus* strains could reflect the differences in the MIC [39,42]. Indeed, differences in the membrane composition between antibiotic-sensitive and resistant strains of *S. aureus* were highlighted in the literature [43].

Biofilms represent complex microbial communities attached to a surface and embedded in an exopolysaccharide matrix. Biofilm producer *S. aureus*, especially the MRSA strain, represents a great risk to the health of patients with wounds, with an increased risk of mortality and morbidity [44,45]. Thus, investigating the antibiofilm activity is crucial in examining bioactive agents to prepare antimicrobial wound dressing. Biosurfactants, including RLs, can affect the initial attachment on different surfaces and inhibit biofilm formation [46,47]. Moreover, RLs are also able to disrupt preformed biofilms [18]. Herein, we demonstrated that M15RL has strong antibiofilm activity, being able to interfere in all the steps of the biofilm lifecycle. In addition, it can also penetrate a mature biofilm, killing bacteria inside. Penetrating the biofilm is one of the main challenges of antibiotics. M15RL showed a high penetration ratio that could be helpful in its possible future utilization as an additive for wound dressings.

As cell membranes represent the main targets of M15RL bioactivity, we utilized red blood cells to evaluate their toxicity on eukaryotic cells. Results showed 50% of hemolysis at the highest tested concentration, while no significant differences with the control were found at MIC and below. This indicated high selectivity for bacterial membranes. Moreover, recently we have demonstrated that M15RL has no toxicity against the human keratinocyte line HaCaT and African green monkey kidney Vero cells [25].

As proof of concept for wound healing application, M15RL-loaded cotton swabs were prepared and tested against *S. aureus* 6538. The coated swabs efficiently inhibited 99.99% of the bacterial load at the lowest amount tested, eradicating the whole bacterial population at the highest. This demonstrates that the swabs could be coated and functionalized with M15RL. Recently, we achieved similar results against enveloped viruses, proving that plastic surfaces can be functionalized with M15RL to inactivate members of the *Herpesviridae* and *Coronaviridae* families [25]. Even if RLs were recently successfully utilized to functionalize medical-grade silicon to counteract *Staphylococcus* spp. biofilms, to the best of our knowledge, this is the first time that RLs have been utilized to functionalize cotton swabs [48,49,50,51]. Moreover, we demonstrated that the functionalized cotton swabs remained active after 3 months of storage at RT (data not shown).

Resistance to ranges of pH and temperature is crucial for industrial applications. Despite being widely reported for their stability, it was recently proved that RLs are rapidly degraded at low pH values [52]. However, the authors demonstrated that the degradation led to rearrangements in the mixture and the formation of new RLs congeners, resulting in an equal biosurfactant activity. Here, we also proved that the antibacterial activity is not affected by high temperatures and pH variation. In this optic, we performed resistance tests to both parameters, focalizing our attention on the antimicrobial activity. In particular, we monitored the stability at 80 °C for up to 24 h and at 121 °C for 20 and 40 min. Then, we recorded the antimicrobial stability of M15RL at pH values ranging from 4.0 to 10.0 for two months. In both cases, we achieved very promising results.

Finally, we also assessed the antimicrobial activity at pH values ranging from 5.0 to 9.0. Correlating the antimicrobial activity to the pH value is essential in wound healing, as open wounds exhibit pH values in the range of 6.5 to 8.5, while chronic wounds have pH levels from 7.5 to 8.5 [53]. Our results showed that the antimicrobial activity of M15RL increases at pH 5.0, and while still remaining active, this decreases at a pH higher than 7.4. The results are in agreement with the literature. Indeed, a recent study demonstrated that the antimicrobial activity against *Bacillus cereus* ATCC 33018 of a commercial mixture of RLs is pH-dependent [39]. Therefore, the different prevailing RLs forms at a certain pH could address the differences in the antimicrobial activities. In fact, at a pH lower than the pKa, the polar groups are protonated, and the nonionic form is predominant. Otherwise, when pH is higher than the pKa, the carboxylic groups are deprotonated, and the RLs are negatively charged. In this condition, due to electrostatic repulsion, RLs cannot interact with cell membranes [39,54].

## 5. Conclusions

In this study, we demonstrated that the RLs produced by the Antarctic bacterium *P. gessardii* M15 are able to kill several clinically isolated strains of *S. aureus*, particularly MRSA and VRSA. We also proved their ability to penetrate and kill bacteria inside a mature biofilm, and then we built a proof of concept to demonstrate their ability to functionalize textiles. These activities, together with the proven resistance to harsh conditions that could be encountered during industrial processes and in infection sites, allow us to imagine their possible applications as additives in wound dressings for chronic wounds.

However, a limit of this study is that our findings are restricted to in vitro models. Therefore, in vivo wound healing studies could be useful to validate our results and may represent the next step of this work.

## Figures and Tables

**Table 1:** Rhamnolipid composition of M15RL, MDRL, and DDRL mixtures.

*R*_t_ (min.)	Rhamnolipids ^1^	M15RL (%) ^2^	MDRL (%) ^2^	DDRL (%) ^2^	Notes
6.1	Rha-Rha-C10	-	0.6	1	
7.8	Rha-C10	-	1.6	1.2	
12.5	Rha-Rha-C11	-	0.5	0.2	
14.5	Rha-C11	-	1.3	0.2	
14.8	Rha-C12:1	0.2	-	-	
17.7	Rha-C12	0.2	-	-	
23.3	Rha-Rha-C8-C9 Rha-Rha-C9-C8	-	-	0.1	
25.0	Rha-Rha-C12:1-C10;O Rha-Rha-C10-C12:1;O	-	-	0.5	
25.5	Rha-C14	0.3	-	-	
26.7	Rha-Rha-C8-C10 Rha-Rha-C10-C8	-	1.8	1.2	
29.5	Rha-Rha-C9-C10 Rha-Rha-C10-C9 Rha-Rha-C8-C11	-	0.1	0.9	
30.1	Rha-C8-C10 Rha-C10-C8	4.2	6.9	1.3	in M15RL, only Rha-C8-C10 is present
31.4	Rha-C10;O-C13;O	-	-	0.7	
33.1	Rha-Rha-C10-C10 Rha-Rha-C8-C12	-	15.2	19.6	Rha-Rha-C8-C12 present only in DDRL
33.7	Rha-C10-C9 Rha-C8-C11	0.7	0.3	0.2	in M15RL, different isomers are present, i.e., Rha-C8-C11 and Rha-C9-C10
34.1	Rha-C12:1-C8 Rha-C10:1-C10 Rha-C10-C10:1 Rha-C8-C12:1	0.5	3.4	0.5	in M15RL, Rha-C10-C10:1 is missing
35.8	Rha-Rha-C10-C11 Rha-Rha-C11-C10	-	0.4	5.1	
36.3	Rha-Rha-C12:1-C10 Rha-Rha-C10-C12:1	-	3.3	6.9	
37.5	Rha-C10-C10 Rha-C8-C12	19.3	35.0	20.8	in M15RL, there is also Rha-C12-C8
39.1	Rha-Rha-C12-C10 Rha-Rha-C10-C12	-	7.7	18.2	
40.8	Rha-C10-C11 Rha-C11-C10	3.8	0.4	1.1	in M15RL, there is also Rha-C12-C9
41.6	Rha-C12:1-C10 Rha-C10-C12:1 Rha-C8-C14:1	29.8	8.7	4.7	in M15RL, Rha-C8-C14:1 is missing
42.2	Rha-Rha-C12-C11	-	-	0.7	
41.5 42.9	Rha-Rha-C14:1-C10 Rha-Rha-C10-C14:1 Rha-Rha-C12:1-C12 Rha-Rha-C12-C12:1	-	0.6	2.9	
44.6	Rha-C12-C10 Rha-C10-C12	29.8	10.0	8.3	
45.6	Rha-C13:1-C10 Rha-C12:1-C11	0.7	-	-	
45.7	Rha-Rha-C14-C10 Rha-Rha-C12-C12 Rha-Rha-C10-C14	-	0.9	3.3	
46.1	Rha-C12:1-C12:1	1.2	-	-	
47.4 49.0	Rha-C14:1-C10 Rha-C10-C14:1 Rha-C12:1-C12 Rha-C12-C12:1	5.6	0.9	0.2	in M15RL, Rha-C10-C14:1 is missing
48.2	Rha-C13-C10 Rha-C12-C11 Rha-C11-C12	0.9	-	-	
52.4	Rha-C14-C10 Rha-C10-C14 Rha-C12-C12	2.4	0.5	0.1	in M15RL, Rha-C10-C14 is missing
54.2	Rha-C16:1-C10 Rha-C14:1-C12	0.4	-	-	

^1^ Rha is the abbreviation for rhamnose. Fatty acyl chains are indicated as C#: N; O, where C# is the number of carbon atoms, N is the number of double-bond equivalents, and O is the number of additional oxygen atoms linked to the hydrocarbon chain. ^2^ Relative abundances.

**Table 2:** Antimicrobial activity of M15RL, MDRL, and DDRL against *S. aureus* strains.

		M15RL		MDRL		DDRL	
	*S. aureus* Strains	MIC (µg/mL)	MBC (µg/mL)	MIC (µg/mL)	MBC (µg/mL)	MIC (µg/mL)	MBC (µg/mL)
	*S. aureus* 6538	12.5	12.5	50	100	50	50
	*S. aureus* 6538p	25	25	100	-	100	-
Clinical isolates	MRSA	25	25	>100	-	>100	-
	MSSA	25	50	100	-	100	-
	β-LPSA	12.5	12.5	50	100	>100	-
	QRSA	25	50	100	-	>100	-
	VRSA	50	50	>100	-	>100	-
	MLSB	12.5	12.5	50	100	100	-

## Data Availability

Not applicable.

## Supplementary Materials

The following supporting information can be downloaded at: https://www.mdpi.com/article/10.3390/pharmaceutics15020700/s1, Figure S1: Total ion chromatogram of the M15RL mixture, Figure S2: Total ion chromatogram of the MDRL mixture, Figure S3: Total ion chromatogram of the DDRL mixture, Figure S4: HR ESI-MS2 spectrum of Rha-C10-C10, Figure S5: MRSA antibiogram, Figure S6: MSSA antibiogram, Figure S7: β—LPSA antibiogram, Figure S8: QRSA antibiogram, Figure S9: VRSA antibiogram, Figure S10: MLSB antibiogram, Figure S11: M15RL antimicrobial stability to different pH levels.
