# Supplementary material for: Evaluation of Antimicrobial Properties and Potential Applications of Pseudomonas gessardii M15 Rhamnolipids towards Multiresistant Staphylococcus aureus
Source: Pharmaceutics. 2023 Feb 19;15(2):700. doi: 10.3390/pharmaceutics15020700 (PMC9958974; doi:10.3390/pharmaceutics15020700)
Supplement: Supplementary file 1 [file pharmaceutics-15-00700-s001.zip › pharmaceutics-2211664-supplementary.pdf]

Antimicrobial properties of *Pseudomonas gessardii* M15 rhamnolipids towards multiresistant *Staphylococcus aureus* and potential applications

Carmine Buonocore [1,2,†], Rosa Giugliano [3,†], Gerardo Della Sala [1], Fortunato Palma Esposito [1], Pietro Tedesco [1], Veronica Folliero [3], Massimiliano Galdiero [3], Gianluigi Franci [4,*] and Donatella de Pascale [1,*]

[1] Department of Ecosustainable Marine Biotechnology Stazione Zoologica Anton Dohrn, Via Ammiraglio Acton, 55, 80133 Naples, Italy
[2] Institute of Biochemistry and Cell Biology, National Research Council, 80131 Naples, Italy
[3] Department of Experimental Medicine, University of Campania "Luigi Vanvitelli", 80138 Naples, Italy
[4] Department of Medicine, Surgery and Dentistry "Scuola Medica Salernitana", University of Salerno, 84081 Baronissi, Italy
* Correspondence: gfranci@unisa.it (G.F.); donatella.depascale@szn.it (D.d.P.)
† These authors contributed equally to this work.

Contents 2

Spectral data of rhamnolipids mixtures 3
Figure S1. Total ion chromatogram of the M15RL mixture 3
Figure S2. Total ion chromatogram of the MDRL mixture 3
Figure S3. Total ion chromatogram of the DDRL mixture 4
Figure S4. HR ESI-MS2 spectrum of Rha-C10-C10 4
Antibiograms of the *Staphylococcus aureus* strains 5
Figure S5: MRSA antibiogram 5
Figure S6: MSSA antibiogram 6
Figure S7: β—LPSA antibiogram 7
Figure S8: QRSA antibiogram 8
Figure S9: VRSA antibiogram 9
Figure S10: MLSB antibiogram 10
M15RL stability to pH 11
Figure S11: M15RL antimicrobial stability to different pH 11

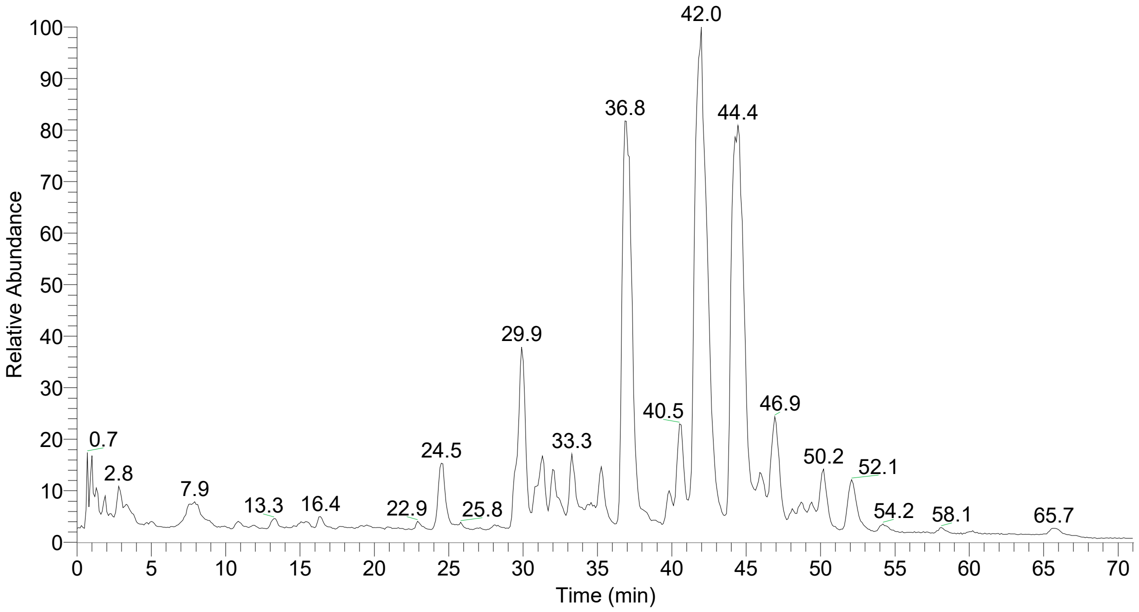

Figure S1. Total ion chromatogram of the M15RL mixture.

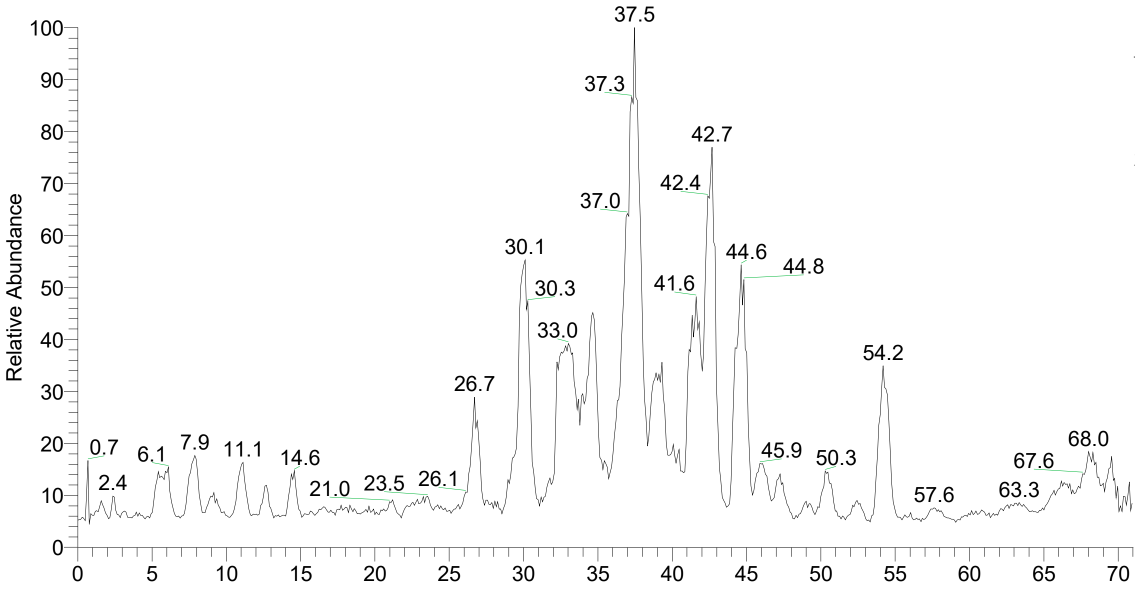

Figure S2. Total ion chromatogram of the MDRL mixture.

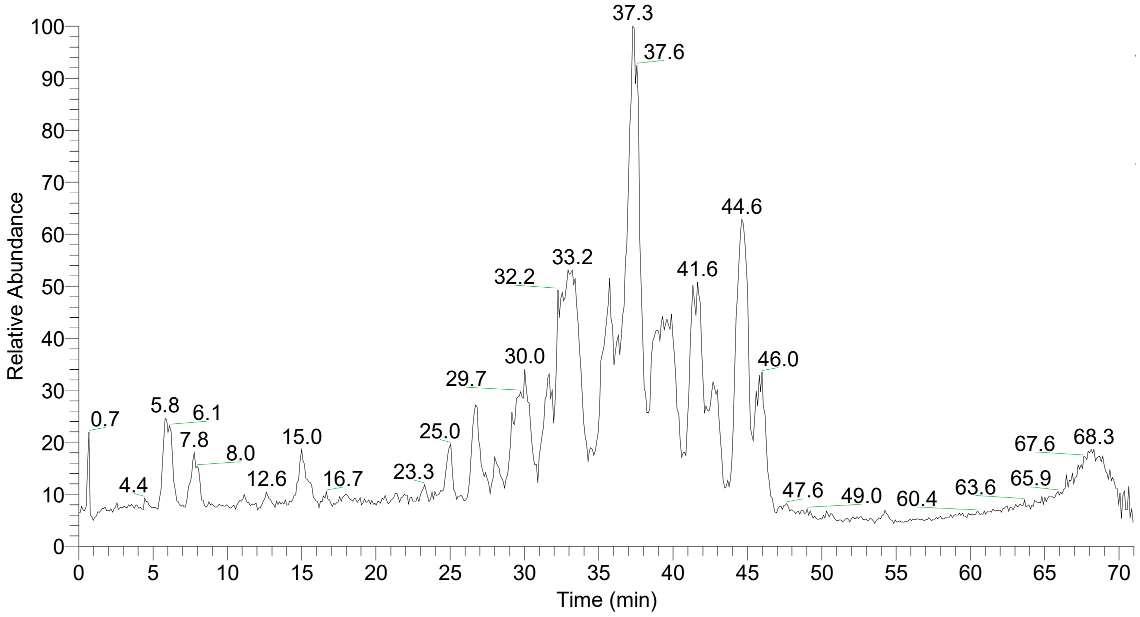

Figure S3. Total ion chromatogram of the DDRL mixture.

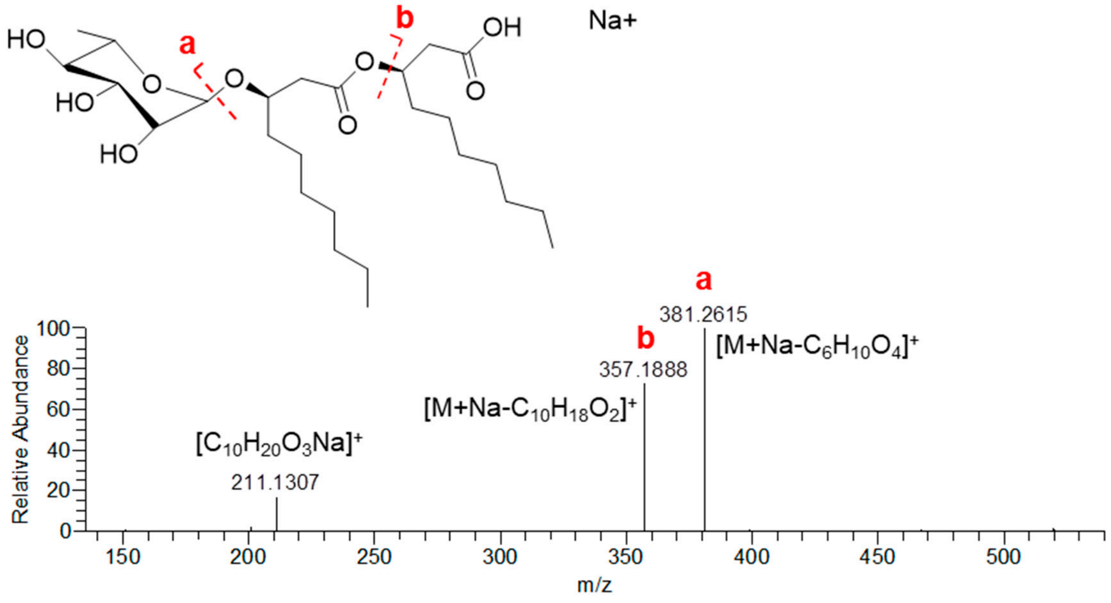

Figure S4. HR ESI-MS2 spectrum of the $[M+Na]^+$ ion of Rha-C10-C10 ($C_{26}H_{48}O_9Na$, *m/z* 527.3199 – Δppm 1.6)

St. AUREUS MRSA
3/12/15

SERVIZIO DI MICROBIOLOGIA

Cliente bioMerieux: S U N
N. sistema: 1498

Lab report

Stampato 27-nov-2015 11:44 CET
Stampato da: LabAdmin

Nome paziente: [redacted]

*** Allerta applicato ***

ID paziente: 52187

Gruppo di isolati: 52187.11-1

Tipo di card: AST-P632 Test dello strumento: 00000B4D4CDB (1498)

Quantità organismo:

Commenti:	ATTENZIONE CEPPO MRSA
	ATTENZIONE CEPPO MRSA

Informazioni sull'identificazione				
Microrganismo selezionato	Staphylococcus aureus			
	Immesso:	25-nov-2015 10:33 CET	Da:	labadmin

Messaggi di analisi:

Un test ICR positivo indica resistenza inducibile ai macrolidi, licosamidi e streptogramina tipo B. Tale isolato si presume resistente alla clindamicina che tuttavia potrebbe essere efficace in alcuni casi.

Resistenza a basso livello - una MIC di 2, 4, 32, 64 per la mupirocina rappresenta lintero range intermedio (2-256).

Informazioni sull'antibiogramma	Card:	AST-P632	Numero di lotto: 732366110		Scade:	22-dic-2016 12:00 CET
	Completato:	25-nov-2015 20:07 CET	Stato:	Finale	Tempo di analisi:	10,00 ore

Antimicrobico	MIC	Interpretazione	Antimicrobico	MIC	Interpretazione
Cefoxitina screening	POS	+	Teicoplanina	<= 0,5	S
Benzilpenicillina	> 0,25	R	Vancomicina	<= 0,5	S
Oxacillina	> 2	R	Tetraciclina	<= 1	S
Gentamicina	> 8	R	Tigeciclina	<= 0,12	S
Levofloxacina	> 4	R	Fosfomicina	> 64	R
Resistenza inducibile alla Clindamicina	POS	+	Acido fusidico	<= 0,5	S
Eritromicina	> 4	R	Mupirocina	<= 2	S
Clindamicina	0,25	*R	Rifampicina	> 2	R
Linezolid	2	S	Trimetoprim/Sulfametossazolo	20	S
Daptomicina	0,25	S			

+= Antibiotici dedotti *= Modificato AES **= Modificato utente

Versione di VITEK 2 Systems installata: 07.01
Linea guida interpretazione MIC: EUCAST 2015_MICETI

Linea guida interpretazione terapeutica: Copia di EUCAST-based_2015

Nome del set di parametri AES: EUCAST_2015_MICETI

Ultima modifica parametri AES: 2-nov-2015 14:13 CET

Pagina 1 di 2

Figure S5. Antibiogram of the MRSA strain

[illegible]
NOS RO S. AUREUS
13/2/15 MSSA

A.O.U. SUN - S.C. BATTERIOLOGIA CLINICA

Cliente bioMerieux:
N. sistema:

Lab report

Stampato 18-nov-2014 11:07 CST
Stampato da: labadmin

Nome paziente [redacted] 24/3/92

ID paziente: 50574

*** Allerta applicato ***

Gruppo di isolati: 50574 11-1

Organismo selezionato: Staphylococcus aureus

Commenti:	ESEGUIRE TEST CEFINASI

Informazioni sull'identificazione			
Microrganismo selezionato	Staphylococcus aureus		
	Immesso: 7-nov-2014 11:46 CST	Da:	labadmin
Messaggi di analisi:			

Informazioni sull'antibiogramma	Card: AST-P632		Numero di lotto: 732323640	Scade: 24-ott-2015 13:00 CDT	
	Completato: 7-nov-2014 20:08 CST		Stato: Finale	Tempo di analisi: 8,50 ore	
**Antimicrobico**	**MIC**	**Interpretazione**	**Antimicrobico**	**MIC**	**Interpretazione**
Cefoxitina screening	NEG	-	Teicoplanina	<= 0,5	S
Benzilpenicillina	0,06	S	Vancomicina	<= 0,5	S
Oxacillina	<= 0,25	S	Tetraciclina	<= 1	S
* Gentamicina	<= 0,5	S	Tigeciclina	<= 0,12	S
Levofloxacina	<= 0,12	S	Fosfomicina		
Resistenza inducibile alla Clindamicina	NEG	-	Acido fusidico	<= 0,5	S
Eritromicina	0,5	S	Mupirocina	<= 2	
Clindamicina	0,25	S	Rifampicina	<= 0,03	S
Linezolid	2	S	Trimetoprim/Sulfametossazolo	<= 10	S
Daptomicina	0,5	S			

+= Antibiotici dedotti *= Modificato AES **= Modificato utente

Conclusioni AES:		Ultima modifica: 8-apr-2014 09:18 CDT	Set di parametri: EUCAST2014+MICETI vers. 5.04
Livello di affidabilità:	Coerente		
Fenotipo:	MACROLIDI/LINCOSAMIDI/STREPTOGRAMINE	RESISTENTE ALLE STREPTOGRAMINE (SGA-SGB)	

Azione	Nome (ID utente)	Data/Ora	Commento
Revisionato da:	(labadmin)	10-nov-2014 09:08 CST	

Versione di VITEK 2 Systems installata: 05.04
Linea guida interpretazione MIC: EUCAST_MOD_2014 Linea guida interpretazione terapeutica: EUCAST-based_MOD_2014
Nome del set di parametri AES: EUCAST2014+MICETIvers. 5.04 Ultima modifica parametri AES: 8-apr-2014 09:18 CDT

Pagina 1 di 1

Figure S6. Antibiogram of the MSSA strain

st. aureus β
3/12/15

Cliente bioMerieux:
N. sistema:

Lab report

Stampato 18-nov-2014 11:06 CST
Stampato da: labadmin

Nome paziente: [redacted] 31/3/89
Gruppo di isolati: 52279-1

ID paziente: 52279.10

Organismo selezionato: Staphylococcus aureus

Commenti:	

Informazioni sull'identificazione			
Microrganismo selezionato	Staphylococcus aureus		
	Immesso: 23-ott-2014 11:19 CDT	Da:	labadmin
Messaggi di analisi:			

Informazioni sull'antibiogramma	Card:	AST-P632	Numero di lotto: 732323640	Scade:	24-ott-2015 13:00 CDT
	Completato:	23-ott-2014 17:48 CDT	Stato: Finale	Tempo di analisi:	8,25 ore
**Antimicrobico**	**MIC**	**Interpretazione**	**Antimicrobico**	**MIC**	**Interpretazione**
Cefoxitina screening	NEG	-	Teicoplanina	<= 0,5	S
Benzilpenicillina	>= 0,5	R	Vancomicina	1	S
Oxacillina	0,5	S	Tetraciclina	<= 1	S
Gentamicina	4	R	Tigeciclina	<= 0,12	S
Levofloxacina	0,25	S	Fosfomicina		
Resistenza inducibile alla Clindamicina	NEG	-	Acido fusidico	<= 0,5	S
Eritromicina	>= 8	R	Mupirocina	<= 2	
Clindamicina	0,25	S	Rifampicina	<= 0,03	S
Linezolid	2	S	Trimetoprim/Sulfametossazolo	<= 10	S
Daptomicina	1	S			

+= Antibiotici dedotti *= Modificato AES **= Modificato utente

Conclusioni AES:	Ultima modifica: 8-apr-2014 09:18 CDT	Set di parametri:	EUCAST2014+MICETIvers. 5.04
Livello di affidabilità:	Coerente		

Azione	Nome (ID utente)	Data/Ora	Commento
Revisionato da:	(labadmin)	3-nov-2014 12:13 CST	

Versione di VITEK 2 Systems installata: 05.04
Linea guida interpretazione MIC: EUCAST_MOD_2014 Linea guida interpretazione terapeutica: EUCAST-based_MOD_2014
Nome del set di parametri AES: EUCAST2014+MICETIvers. 5.04 Ultima modifica parametri AES: 8-apr-2014 09:18 CDT

Pagina 1 di 1

Figure S7. Antibiogram of the β—LPSA strain

REFERTO - FINALE

Pagina 1/1
26/07/2017 10:08:46

Nome paziente: [redacted] **ID paziente:51883.07**
Data di nascita: Sesso paziente:Non specificato

N accesso: **51883.07**
Tipo campione: CATETERE VENOSO CENTRALE
Reparto ospedaliero: 7301 TER INTENSIVA NEONATALE
Data ricezione: 21/07/2017 09:49:51

Nome organismo **Commenti**
1 STAAUE Staphylococcus aureus

Marker di resistenza
1 STAIML Fenotipo MLSb inducibile di Staphylococcus

Antibiotico	STAAUE MIC/*Conc.*	SIR
Acido fusidico	<=0,5	S
Ampicillina		R
Ceftaroline	0,5	S
Ciprofloxacina	>4	R
Clindamicina		R
Daptomicina	<=0,5	S
Eritromicina	>2	R
Fosfomicina c/G6P	<=16	S
Gentamicina	2	R
Linezolid	2	S
Moxifloxacina	>1	R
Mupirocina alto livello	<=256	S
Oxacillina	0,5	S
Penicillina G	>0,25	R
Teicoplanina	<=0,5	S
Tetraciclina	<=0,5	S
Tigeciclina	<=0,25	S
Trimetoprim-sulfametoxazolo	<=1/19	S
Vancomicina	1	S

Firma: ______________________

S. aureus CHIN3

Figure S8. Antibiogram of the QRSA strain

NOS R5
5/12/16

SERVIZIO DI MICROBIOLOGIA

Cliente bioMerieux: S U N
N. sistema: 1498

Lab report

Stampato 4-dic-2014 10:27 CST
Stampato da: labadmin

Nome paziente: [redacted] 28/02/82

*** Allerta applicato ***

ID paziente: 50211

Gruppo di isolati: 50211.12-2

Organismo selezionato: Staphylococcus aureus

**Commenti:**	Attenzione Stafilococco Teicoplanina Resistente Attenzione Stafilococco Vancomicina Resistente

**Informazioni sull'identificazione**			
**Microrganismo selezionato**	Staphylococcus aureus		
	**Immesso:** 3-dic-2014 10:55 CST	**Da:**	labadmin
**Messaggi di analisi:**			

Informazioni sull'antibiogramma	**Card:**	AST-P632	**Numero di lotto:** 732323640	**Scade:**	24-ott-2015 13:00 CDT
	**Completato:**	3-dic-2014 22:29 CST	**Stato:** Finale	**Tempo di analisi:**	12,25 ore

Antimicrobico	MIC	Interpretazione	Antimicrobico	MIC	Interpretazione
Cefoxitina screening	NEG	-	Teicoplanina	<= 0,5*	*R
Benzilpenicillina	>= 0,5	R	Vancomicina	>= 32	R
Oxacillina	<= 0,25	S	Tetraciclina	<= 1	S
Gentamicina	>= 16	R	Tigeciclina	<= 0,12	S
Levofloxacina	1	S	Fosfomicina		
Resistenza inducibile alla Clindamicina	NEG	-	Acido fusidico	<= 0,5	S
Eritromicina	1	S	Mupirocina	<= 2	
Clindamicina	>= 4	R	Rifampicina	<= 0,03	S
Linezolid	2	S	Trimetoprim/Sulfametossazolo	<= 10	S
Daptomicina	0,25	S			

+= Antibiotici dedotti *= Modificato AES **= Modificato utente

Conclusioni AES:	**Ultima modifica:** 8-apr-2014 10:22 CDT	**Set di parametri:** EUCAST2014+MICET lvers. 5.04
**Livello di affidabilità:**	Coerente con la correzione	
**Fenotipo:**	GLICOPEPTIDI	VRSA

Versione di VITEK 2 Systems installata: 05.04
Linea guida interpretazione MIC: EUCAST_MOD_2014
Linea guida interpretazione terapeutica: EUCAST-based_MOD_2014
Nome del set di parametri AES: EUCAST2014+MICETlvers. 5.04
Ultima modifica parametri AES: 8-apr-2014 10:22 CDT

Pagina 1 di 1

[redacted]

Figure S9. Antibiogram of the VRSA strain

3/12/15

Cliente bioMerieux: Rapporto grafico Microbiologia Stampato 20-ott-2014 08.43 CDT

Nome paziente: [redacted]
Posizione:
ID lab.: 51119

ID paziente:
Medico:
Numero di isolato: 1

Organismo selezionato: Staphylococcus aureus

Prelevato:

Origine:

Commenti:	

Informazioni sull'antibiogramma	Tempo di analisi:	9,50 ore		Stato:	Finale
**Antimicrobico**	**MIC**	**Interpretazione**	**Antimicrobico**	**MIC**	**Interpretazione**
Cefoxitina screening	NEG	-	Daptomicina	1	S
Benzilpenicillina	>= 0,5	R	Teicoplanina	<= 0,5	S
Oxacillina	<= 0,25	S	Vancomicina	<= 0,5	S
Gentamicina	<= 0,5	S	Tetraciclina	<= 1	S
Levofloxacina	0,25	S	Tigeciclina	<= 0,12	S
* Resistenza inducibile alla Clindamicina	NEG	-	Acido fusidico	<= 0,5	S
* Eritromicina	>= 8	R	Rifampicina	<= 0,03	S
Clindamicina	>= 4	R	Trimetoprim/Sulfametossazolo	<= 10	S
Linezolid	2	S			

+= Antibiotici dedotti *= Modificato AES **= Modificato utente

Conclusioni di AES		
Affidabilità:	Coerente	
Fenotipo:	MACROLIDI/LINCOSAMIDI/STREPTOGRAMINE	MLSB+SA COSTITUTIVO

Pagina 1 di 1

Figure S10. Antibiogram of the MLSB strain

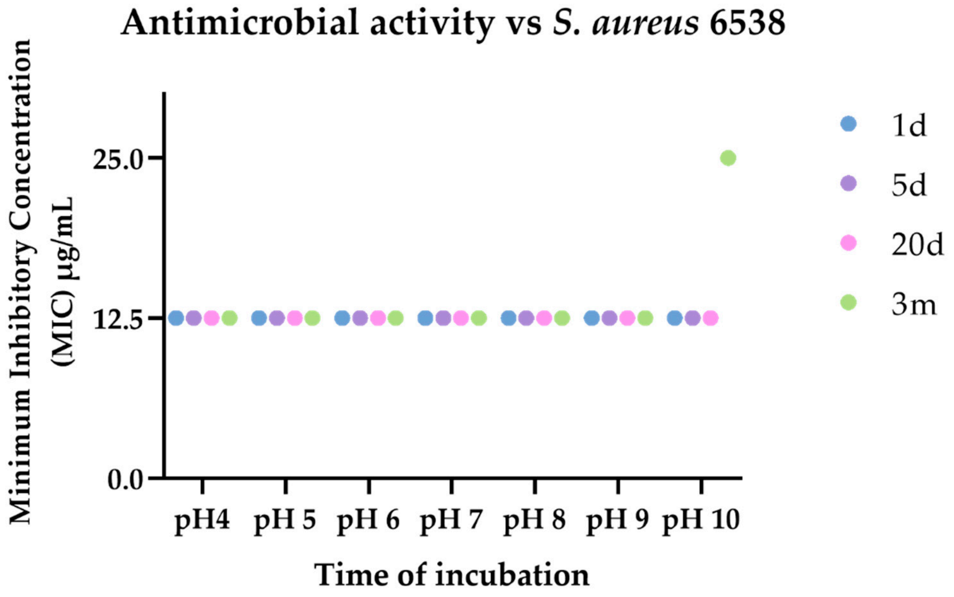

Figure S11. M15RL antimicrobial stability to different pH. M15RL was incubated at different pH for 3 months and tested for antimicrobial activity against *S. aureus* 6538 at different intervals.
